# Comparative genomics and association analysis identifies virulence genes of *Cercospora sojina* in soybean

**DOI:** 10.1186/s12864-020-6581-5

**Published:** 2020-02-19

**Authors:** Xin Gu, Junjie Ding, Wei Liu, Xiaohe Yang, Liangliang Yao, Xuedong Gao, Maoming Zhang, Shuai Yang, Jingzhi Wen

**Affiliations:** 1 Department of Plant Protection, College of Agriculture, Northeast Agricultural University, Harbin, China; 2 Jiamusi Branch of Heilongjiang Academy of Agricultural Sciences, Jiamusi, China; 3 Potato Research Institute, Heilongjiang Academy of Agricultural Sciences, Harbin, 150086 China

**Keywords:** *Cercospora sojina*, Functional annotation, Gene prediction, Genome sequencing, Genome-wide association analysis

## Abstract

**Background:**

Recently, a new strain of *Cercospora sojina* (Race15) has been identified, which has caused the breakdown of resistance in most soybean cultivars in China. Despite this serious yield reduction, little is known about why this strain is more virulent than others. Therefore, we sequenced the Race15 genome and compared it to the Race1 genome sequence, as its virulence is significantly lower. We then re-sequenced 30 isolates of *C. sojina* from different regions to identifying differential virulence genes using genome-wide association analysis (GWAS).

**Results:**

The 40.12-Mb Race15 genome encodes 12,607 predicated genes and contains large numbers of gene clusters that have annotations in 11 different common databases. Comparative genomics revealed that although these two genomes had a large number of homologous genes, their genome structures have evolved to introduce 245 specific genes. The most important 5 candidate virulence genes were located on Contig 3 and Contig 1 and were mainly related to the regulation of metabolic mechanisms and the biosynthesis of bioactive metabolites, thereby putatively affecting fungi self-toxicity and reducing host resistance. Our study provides insight into the genomic basis of *C. sojina* pathogenicity and its infection mechanism, enabling future studies of this disease.

**Conclusions:**

Via GWAS, we identified five candidate genes using three different methods, and these candidate genes are speculated to be related to metabolic mechanisms and the biosynthesis of bioactive metabolites. Meanwhile, Race15 specific genes may be linked with high virulence. The genes highly prevalent in virulent isolates should also be proposed as candidates, even though they were not found in our SNP analysis. Future work should focus on using a larger sample size to confirm and refine candidate gene identifications and should study the functional roles of these candidates, in order to investigate their potential roles in *C. sojina* pathogenicity.

## Background

Frogeye leaf spot (FLS) is caused by *Cercospora sojina* Hara (*C. sojina*) and was first reported in Japan in 1915 [1]. The disease spreads rapidly in susceptible cultivars and is dependent on the interactions of leaf wetness periods and temperature [2]. Recently, FLS has expanded, seriously threatening worldwide soybean production [3]. In 2009 and 2010, the disease spread rapidly throughout the main soybean producing areas in Heilongjiang province in China, causing serious losses to soybean production [4]. Further, the disease has dramatically impacted soybean production in the USA and Argentina [5, 6].

Although the disease can be controlled using pesticides, the planting of resistant cultivars is the preferred disease mitigation strategy. However, the use of resistant soybean cultivars has an obvious drawback in that disease resistance is rapidly lost. The main reason for this is that resistance mechanisms are overcome by the emergence of new *C. sojina* pathotypes [7, 8]. Athow first reported the physiological differentiation of *C. sojina* and identified Race1 and Race2, but 11 U.S. races were subsequently identified using a set of 16 differential cultivars [9, 10]. Additionally, there have been 22 races of *C. sojina* found in Brazil to date [11, 12]. Moreover, *C. sojina* races have undergone rapid evolution and positive selection in the Chinese main soybean production area, with 15 races being reported in Heilongjiang province. EST-SSRs (Expressed Sequence Tag-Simple Sequence Repeats) were analyzed to determine the genotypic structure of these races, and the Race15 strain was found to be genetically close to the Race1 strain, in addition to them having similar pathogenic response types [13]. Among these races, the new Race15 strain is considered to be the dominant race, occurring at a frequency of 36%, more than the previously dominant Race1 strain [13, 14]. This has led to a loss of resistance in many cultivars in Heilongjiang province.

Although there are many races in different soybean production areas of the world, the differential cultivars used in different countries leads to an incompatibility of these different *C. sojina* strains, providing a potential method of identifying the races of this disease. Unfortunately, this method is greatly impacted by the environment, so differential disease-resistant seeds cannot be easily used in different regions. Previously, Li and Hu used Chinese differential cultivars to characterize different races of *C. sojina* from other countries; however, they only found that Race4 in China was the same as Race1 in the US, and that Race3 in China was the same as the Brazilian Race 2, with no other races being similar between regions [15, 16].

In addition to distinguishing races using differential cultivars, there are molecular genetic tools such as AFLPs (Amplified Fragment Length Polymorphisms), SSR markers and SNP markers that can be used to characterize the population diversity of *C. sojina*. *C. sojina* has a high evolutionary potential in that it reproduces both sexually and asexually, allowing it to rapidly overcome host genetic resistance through recombination. Based on the mating type distribution, the sexual reproduction of *C. sojina* was postulated to exist in Arkansas populations, as all six populations evaluated here had high genotypic diversity and significant genetic exchange existed [17]. Clone-corrected data indicated that the proportion of the MAT1–1 idiomorph and the MAT1–2 idiomorph was approximately the same in these areas [18]. Studies like these have been very useful for the large-scale screening of isolates for mating type and for understanding the population dynamics of *C. sojina*. Scientists in the United States, however, have differentiated strains of *C. sojina* from another perspective. They have used SNP markers in the mating type loci of *C. sojina* isolates in order to investigate population diversity and have determined that its resistance genes relate to quinone outside inhibitor (QoI). These reports identified 49 unique SNPs, and the QoI resistance locus was genotyped from 186 isolates, revealing 35 unique genotypes [19]. These data also indicated that *C. sojina* is still evolving with respect to QoI resistance under the pressure of fungicides. This fact explains why, despite soybean producers in the United States using fungicides to control the disease for many years, it is still spreading. A previous study also used AFLP markers to analyze the genetic diversity of 62 *C. sojina* isolates from major soybean producers in the world. The cluster analysis from this work showed that these isolates can be divided into 2 major clusters and 7 sub-clusters. Except for 2 isolates from Georgia, USA, and 2 isolates from China that were clustered together, respectively, none of the others were found to be clustered together. Because of the abundant genetic diversity shown by this study, broad-spectrum disease resistance should be the main objective of disease resistance breeding [20].

The genomes of Race1 [21] (China), FLS21 [3] (USA), and S9 [19] (USA) have recently been sequenced. The genome size of the Chinese strain was found to be significantly larger than the sizes of the American FLS21 and S9 strains. The previous studies imply that *C. sojina* can flexibly adapt to its environment and host changes. Most of the repeats in the *C. sojina* genome are less than 100 bp in size and they display distinct repeat organization properties compared with the other pathogen members of the genus *Mycosphaerella* [22]. In recent years, we have found that the varieties of soybean resistant to Race1 have gradually lost their resistance and a large number of disease spots appear on the leaf surface of infected plants. The isolates were identified as the new Race15 using Chinese differential cultivars. The Race15 separation frequency and virulence were significantly higher than that of Race1 [13]. In 2017, whole genome sequencing of Race1 was completed [21], showing that Race1 lacks any PKS-NRPS hybrids, PKS-like proteins, or dimethylallyl tryptophan synthases. *C. sojina* Race1 also has a large group of potential carbohydrate esterases (CEs), which can catalyze the O- or *N*-deacetylation of substituted saccharides. Numerous pathogenicity-related genes were found via whole genome transcription assays [21]. It is interesting that one of the enriched families is the glycosidehydroxylate GH109 family, which encodes α-*N*-acetylgalactosaminidase. The GH109 family in *C. sojina* has been speculated to overcome lectin-mediated disease resistance in soybean. It may compete with soybean lectin to bind *N*-acetyl galactosamine. Plant lectin binding with it can inhibit hyphae growth and spore germination in several fungi, such as *Penicillia* and *Aspergilli* species [23]. These may account for the differences in virulence between strains. At present, the causes of differences in the virulence of different strains is unknown, and there are few reports on differential virulence genes and intraspecific virulence differentiation of *C. sojina*.

This study is the first to use phenotype-genotype association to prioritize candidate effectors at the genome-wide scale, through the careful matching of virulence profiles from nationwide strain surveys of *C. sojina* in China. It is also the first to use comparative genomic analysis to explore the differences in virulence genes between strains with different virulence. This study provides a basis for further study of the pathogenic mechanisms and molecular mechanisms of disease resistance breeding and also provides a reference for the identification of other fungal virulence genes. Functional studies of these candidate genes would be the next logical step for investigating their potential role in the pathogenicity of *C. sojina*.

## Results

### Virulence evaluation of *Cercospora sojina* isolates

Isolates were collected from major soybean producing areas in northeast China, including 29 isolates from Heilongjiang province and three from Jilin province. Virulence testing showed that the disease index of the isolates ranged from 20.31 to 90.25, and among these the Race15 isolate had the highest virulence and the Race1 isolate had the lowest (Table 1).

**Table 1:** Virulence evaluation of *C. sojina* isolates

Isolate	Disease index	Collection year	Collection location
Tj	60.28 ± 1.99	2015	Tongjiang City, Heilongjiang province (N47°42′25.16″, E132°35′18.60″)
B	42.12 ± 1.03	2000	Beian City, Heilongjiang province (N31°33′51.91″, E104°34′13.44″)
C	59.68 ± 4.27	2000	Yian City, Heilongjiang province (N47°53′41.48″, E125°17′53.66″)
HL2	68.55 ± 7.55	1999	Hailun City, Heilongjiang province (N47°28′11.84″, E126°57′31.72″)
Fj	54.22 ± 7.94	2010	Fujin City, Heilongjiang province (N47°14′34.36″, E132°04′58.55″)
A	42.55 ± 3.79	1999	Mudanj City, Heilongjiang province (N44°31′28.15″, E129°39′29.75″)
HH	30.28 ± 4.95	2016	Heihe City, Heilongjiang province (N50°14′34.73″, E127°28′35.85″)
DH	20.34 ± 4.78	2016	Dunhua City, Jilin province (N43°22′5.27″, E128°13′26.01″)
HN	40.11 ± 3.84	2010	Huanan City, Heilongjiang province (N46°14′1.10″, E130°33′0.08″)
Fj2	60.38 ± 4.09	1999	Fujin City, Heilongjiang province (N47°14′34.36″, E132°04′58.55″)
E	45.28 ± 5.08	1999	Wudalianchi City, Heilongjiang province (N48°31′31.39″, E126°12′56.25″)
Hg	70.21 ± 6.25	2011	Hegang City, Heilongjiang province (N47°25′9.45″, E130°20′24.79″)
HXL	50.28 ± 3.12	2012	Hongxinglong City, Heilongjiang province (N46°44′32.50″, E131°38′42.04″)
KF9	34.28 ± 3.89	2011	Haerbin City, Heilongjiang province (N45°51′50.28″, E126°28′18.64″)
WQ	40.22 ± 2.04	2016	Wangqing City, Jilin province (N43°20′2.07″, E129°47′44.60″)
SB	87.59 ± 2.40	2016	Suibin City, Heilongjiang province (N47°17′33.92″, E131°51′48.82″)
Jh	35.28 ± 4.51	2016	Jiaohe City, Jilin province (N43°43′36.64″, E127°20′41.17″)
BQL	60.23 ± 1.91	2000	Baoquanling City, Heilongjiang province (N47°25′48.83″, E130°31′18.30″)
HL	24.58 ± 2.13	1999	Hailun City, Heilongjiang province (N47°29′54.81″, E126°57′23.72″)
JS	80.57 ± 1.87	2016	Jinshan City, Heilongjiang province (N47°12′20.81″, E128°33′17.60″)
Jx	75.42 ± 4.14	2016	Jixian City, Heilongjiang province (N46°44′0.76″, E131°06′59.73″)
JY	78.58 ± 3.75	2016	Jiayin City, Heilongjiang province (N48°53′21.91″, E130°24′11.09″)
Ks	70.28 ± 6.55	2010	Keshan City, Heilongjiang province (N48°02′7.48″, E125°52′40.22″)
BQL1	71.21 ± 8.49	2010	Baoquanling City, Heilongjiang province (N47°25′48.83″, E130°31′18.30″)
BQL3	70.25 ± 1.97	2016	Baoquanling City, Heilongjiang province (N47°28′5.12″, E130°36′47.49″)
SH	65.58 ± 5.40	2016	Suihua City, Heilongjiang province (N46°39′45.97″, E126°57′49.78″)
HL1	64.21 ± 8.86	2000	Hailun City, Heilongjiang province (N47°22′28.01″, E127°01′31.69)
D	60.25 ± 2.15	1995	QiQihaer City, Heilongjiang province (N47°18′38.77″, E124°12′15.18″)
Fj3	58.59 ± 3.19	2010	Fujin City, Heilongjiang province (N47°22′1.59″, E132°02′3.43″)
JMS	62.35 ± 5.22	2010	Jiamusi City, Heilongjiang province (N46°43′9.49″, E130°40′32.57″)
Race15	90.25 ± 0.87	2015	Jiamusi City, Heilongjiang province (N46°47′25.64″, E130°30′5.63″)
Race1	20.31 ± 7.70	2000	Baoquanling City, Heilongjiang province (N47°28′18.33″, E131°0′6.60″)

### Genome assembly and general characteristics

In total, 601,794 high quality reads were generated by PacBio sequencing, covering 6,038,283,778 bp in total, and having a mean length of 10,033 bp and an N50 length of 13,900 bp. The genome of the Race15 strain of *C. sojina* (40.12 Mb) consisted of 12 curated contigs, with an N50 length of 4.9 Mb. 12,607 coding genes were predicted, at a gene density of approximately 314 genes per Mb (Table 2 and Additional file 1: Table S1). Additionally, non-coding genes were predicted, with 200 tRNA, 2 sRNA and 13 snRNA genes being predicted in the genome of Race15 (Additional file 2: Table S2). A total of 2,140,679 bp of repetitive sequences were identified, representing 5.34% of the Race15 genome. These included DNA transposons, LTR retrotransposons, tandem repeat sequences and other unclassified transposons (Additional file 3: Table S3).

**Table 2:** Genome features of *C. sojina* strains Race15, Race1, FLS 21 and S9

Features	Race15	Race1	FLS 21	S9
Size (bp)	40,115,976	40,836,407	15,477,581	29,949,529
(GC) percentage (%)	53.40	53.12	53.70	53.60
N50 (bp)	4,908,823	1,594,385	˗˗˗	˗˗˗
Contigs	12	62	144	1804
Contigs Max Length (bp)	5,892,303	˗˗˗	˗˗˗	˗˗˗
Gene number (#)	12,607	12,651	5430	8068
Gene total length (bp)	21,192,020	21,035,174	8,401,832	11,859,021
Gene average length (bp)	1681	1663	1547	1470
Gene length/Genome (%)	52.83	51.51	54.28	39.6
Accession	PRJNA508859	PRJNA371568	PRJNA359929	PRJNA82175

### Race15 gene annotation and prediction

**Fig. 1:**
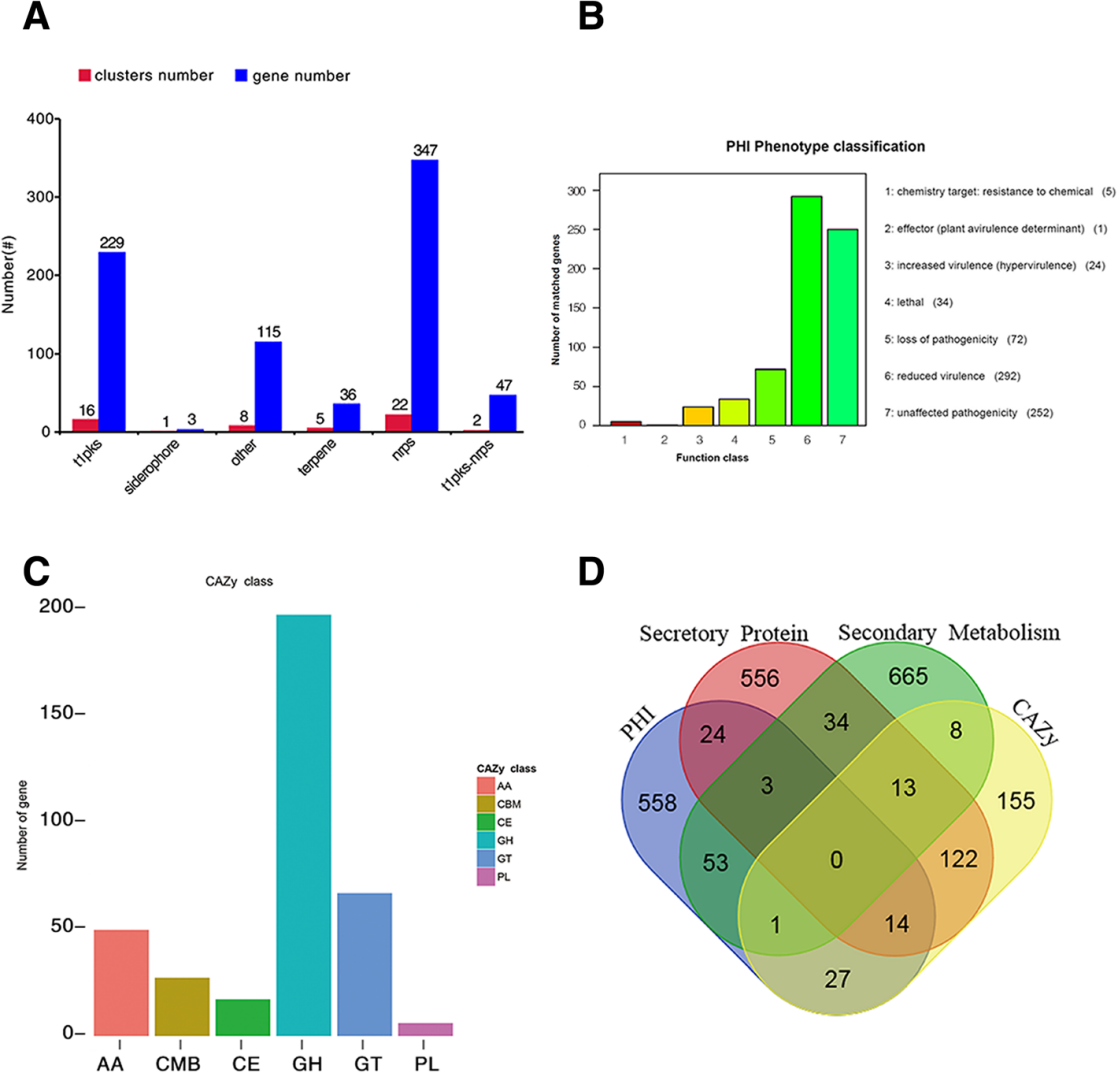
Gene annotation and gene prediction of *C. sojina* Race15. **a** Genes were predicted to be related to Secondary Metabolism. Red bar represents the clusters number, and blue bar represents the number of genes. **b** Genes were annotated and classified in the PHI database. Bars in different color represent different PHI function classes, and lengths represent the number of genes. **c** Genes were annotated and classified in the CAZy database. Bars in different colors represent different CAZy categories, and length represents the number of genes. **d** Venn diagram showing the overlap of PHI-homologues and secretory proteins with secondary metabolites and CAZymes

In order to annotate the function of the predicted genes in the Race15 genome build, seven different databases were used to annotate and predict genes in our Race15 genome build (Additional file 4: Table S4). Previous studies have shown that genes annotated using the PHI and CAZy databases, or predicted as Secretory Protein or Secondary Metabolism, are most likely associated with fungal virulence (Fig. 1d) [24, 25]. A total of 680 genes were annotated and classified using the PHI database (Additional file 5: Table S5). 292 genes were annotated as reduced virulence, and there were 252 genes annotated as unaffected pathogenicity. 72 genes were annotated as pathogenic loss and 34 genes were annotated as lethal factors. 24 genes were annotated as virulence enhanced, 5 genes were annotated as a chemical target: resistant; however, only 1 gene was annotated as effector (plant nontoxic determinants) (Fig. 1b). Successful phytopathogenic fungi can break down and utilize plant cell wall polysaccharides using CAZymes [21]. There were 340 genes annotated in the CAZy database, which could be divided into 5 categories and 1 structural domain. Some genes could be annotated as belonging to two or more classes at the same time. Among these, there were 49 genes annotated as Auxiliary Activities enzymes (AAs), 17 genes annotated as CEs, 195 genes annotated as Glycoside Hydrolases (GHs), 66 genes annotated as Glycosyl Transferases (GTs), 6 genes annotated as Polysaccharide Lyases (PLs), and 27 genes annotated as Carbohydrate-Binding Modules (CBMs) (Fig. 1c).

Pigments are another important group of secondary metabolites used for the successful invasion of pathogens. Previous studies have found that *C. sojina* can produce some grey pigments, that are significantly induced by both starvation and cAMP treatments, suggesting that these pigments may be related to pathogen virulence [21]. A total of 777 genes were predicted to be related to Secondary Metabolism (Fig. 1a), and among these, 16 clusters of 229 genes were predicted as Type I polyketide synthases, 1 cluster of 3 genes was predicted as siderophore and 5 clusters of 36 genes were predicted as Terpene. There were 22 clusters of 347 genes predicted as non-ribosomal peptide synthase (NRPS). There were 2 clusters of 47 genes predicted as t1pks-nrps. In addition, there were 8 clusters of 115 genes predicted as others. Secreted proteins were predicted by Signal P and TMHMM, and the proteins containing signal peptides without obvious transmembrane structure were annotated as secreted proteins. Most phytopathogenic fungi can secrete many proteins and metabolites during the plant–fungi interaction, and these secreted proteins and metabolites play important roles at different infection stages of fungal penetration, colonization, and lesion formation [26, 27]. According to our screening results, 766 genes were predicted as Secretory Proteins.

### Synteny analysis between Race15 and Race1

**Fig. 2:**
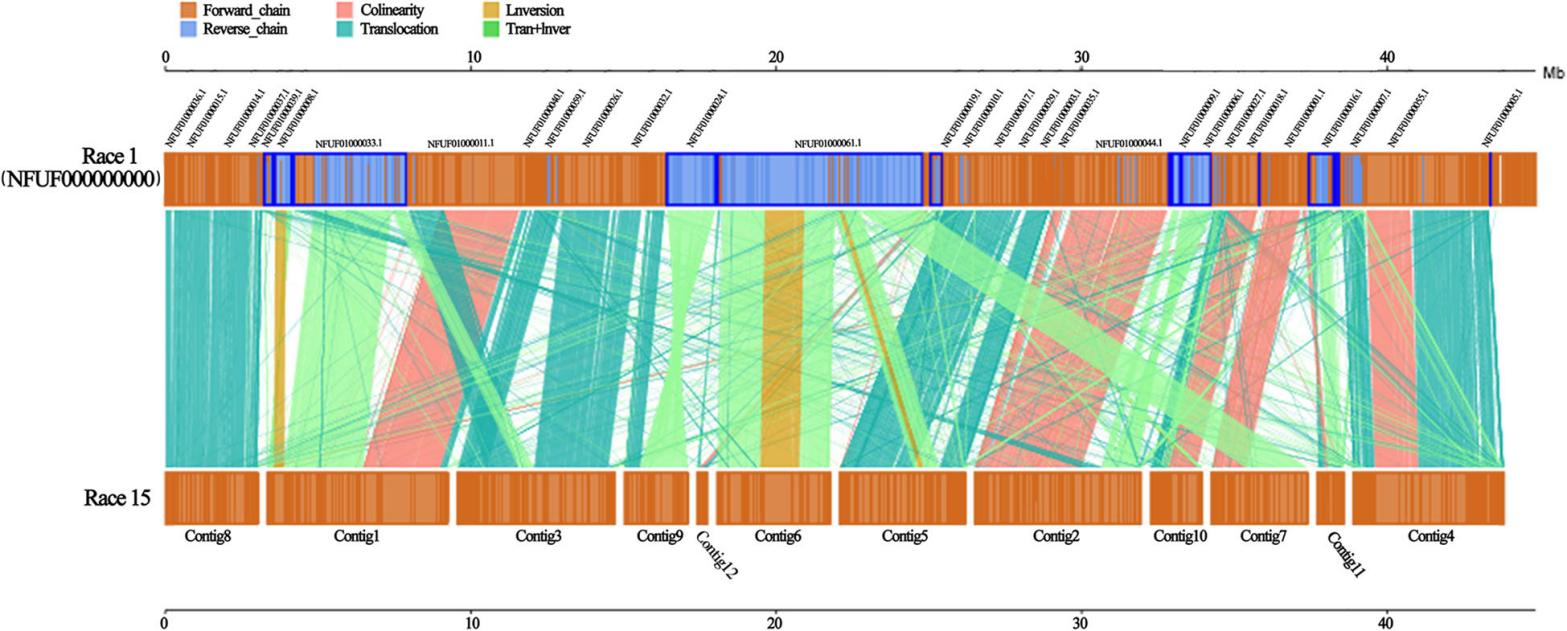
Synteny analysis and core-pan gene analysis of *C. sojina* Race1 with Race15. The top axis represents Race1 and the bottom axis represents Race15. The yellow box in the upper and lower axes represents the forward strand of the genome. The blue box represents the reverse strand of the genome. The color of the link graph between the upper and lower axes indicates the type of comparison

While the Race15 and Race1 genomes largely corresponded, there were various inversion, translocation, and Tran+Inver (translocation and inversion) events that disrupted the otherwise collinear gene order. A comparison of these two strains showed high coverage and synteny. While 93.6% of the regions in the Race15 genome showed synteny with the Race1 strain, only 91.95% of the regions in the Race1 genome showed synteny with Race15 (Additional file 6: Table S6). Although they have good coverage relative to each other, the colinearity was relatively low. Sequence comparisons between the genome assemblies of Race1 and Race15 exhibited colinearity in Contigs 1, 2, 4, 7, 10, and 11. Contigs 3, 5, and 8, however, had large segment translocations. Moreover, a comparison of translocation and inversion in Contig 9 accounted for most of the non-syntenic fragments. Comparison of Contig 6 revealed it was mainly comprised of inversion or Tran+Inver fragments (Fig. 2). Synteny analysis revealed that although the two genomes contained most of the same genes, in the process of evolution they respectively experienced a significant volume of distinct genome structure variation. This resulted in some changes in genomic structures, leading to changes in coding genes, and even changes in functional proteins, especially in non-linear areas.

### Core and orphan gene content

Comparing Race15 and Race1 at the genetic level, we found that the functional differences were caused by the genetic differences. We conducted core-pan gene analysis of Race15 and Race1, with the assumption that the specific genes of Race15 may be related to its increased virulence. Race15 and Race1 were highly homologous. 25,258 genes were clustered into 10,843 clusters by cd-hit, including 10,356 core genes and 487 dispensable genes. There were also many paralogous genes, and these paralogous genes encoded similar functions. The high homology, identity, and coverage between paralogous genes generally passed the threshold for similarity using cd-hit. Therefore, paralogous genes were clustered within the same cluster. Among these clusters, 417 clusters contained more than 3 genes. The largest cluster contained 210 genes. After clustering, homologous genes existing in both samples were removed, and the genes that were present in only one sample were identified as specific genes, Race15 and Race1 had 245 and 274 specific genes, respectively, with the Race15 specific genes possibly being associated with high virulence (Fig. 2 and Additional file 7: Table S7). There were 6 genes annotated in the PHI database, 16 genes predicted as Secretory Protein, 4 genes annotated in the CAZymes database, and 23 genes predicted as secondary metabolic processes. We analyzed these new genes carefully and speculated that they were mainly related to virulence, reduced virulence, unaffected pathogenicity, nrps, t1pks, α-l-fucosidase, β-1,3-glucanase, β-xylanase and other virulence related categories. One of the genes in particular deserved attention, Vtc4 (A07645), which has been shown to be associated with virulence increase in *Cryptococcus neoformans* [28]. It encodes a protein with similarity to the yeast vacuolar transport chaperone for polyphosphate synthesis, and deletion of this gene reduced polyphosphate formation, influenced the transition between yeast and filamentous growth, and attenuated virulence [29]. The genes that were specific to Race15 and not annotated previously could have been missed in our genome annotation. To make up for this shortcoming, we performed a homologous comparison and annotation of Race15 genes with various databases, and annotated Race15 genes as much as possible to facilitate subsequent screening of high-virulence-related genes (Additional file 8: Table S8).

### Genome-wide genotyping of 30 different *C. sojina* isolates

To obtain the sequence variation between different *C. sojina* isolates, Single nucleotide polymorphisms (SNPs) were identified in 30 *C. sojina* isolates from different geographical locations by mapping the sequence reads of each isolate independently to our Race15 reference genome. Of the 30 isolate re-sequencing data, each generated between 1.8 and 7.5 Gb of data. The median aligned read coverage was 42-fold and the minimum and maximum coverage was 11-and 55-fold, respectively. For each isolate, between 23.98 and 97.33% of the sequence reads were mapped to the Race15 reference genome, which covered between 99.05 and 99.68% of the reference genome base. Subsequent resequencing analysis relied mainly on the comparison of data to our reference genome (Additional file 9: Table S9). We noticed two isolates, SB (23.98%) and SH (26.62%), had the lowest percentages of reads aligning to the reference Race15 genome. Therefore, we tried to assemble the sequences that were not aligned, and found they aligned to a species of *Paenibacillus*. However, when we used the SNPs obtained by comparison to the reference genome to construct phylogenetic trees and screen candidate genes, the coverage of the reference genome reached more than 99%, meaning that our results would not be affected by this contaminant species.

Across all the isolates, an average of 12,674 SNPs per isolate was found, covering 0.032% of the reference genome. Except for Race1 (in which we could not identify heterozygous mutations because it was not re-sequenced), out of the 31 isolates, the number of SNPs and homozygous mutations in the KS strain was the largest, at 13,580 and 12,501, respectively. The SNP density of the 31 isolates was between 0.22 and 0.34 relative to the reference genome. The lowest was DH, at 0.22 SNPs per KB, while the SNP density values of A, B and 13 other isolates were all 0.34 SNPs per KB.

Among the 31 isolates, KS had the largest number of non-synonymous (NSY) SNPs at 2998. DH had the lowest number of NSY SNPs, at 1901. In addition, a total of 31,812 SNP sites were identified by merging the SNP sites of the 31 isolates (Additional file 10: Table S10 and Additional file 11: Table S11).

### Phylogenetic analysis of whole-genome SNP data

**Fig. 3:**
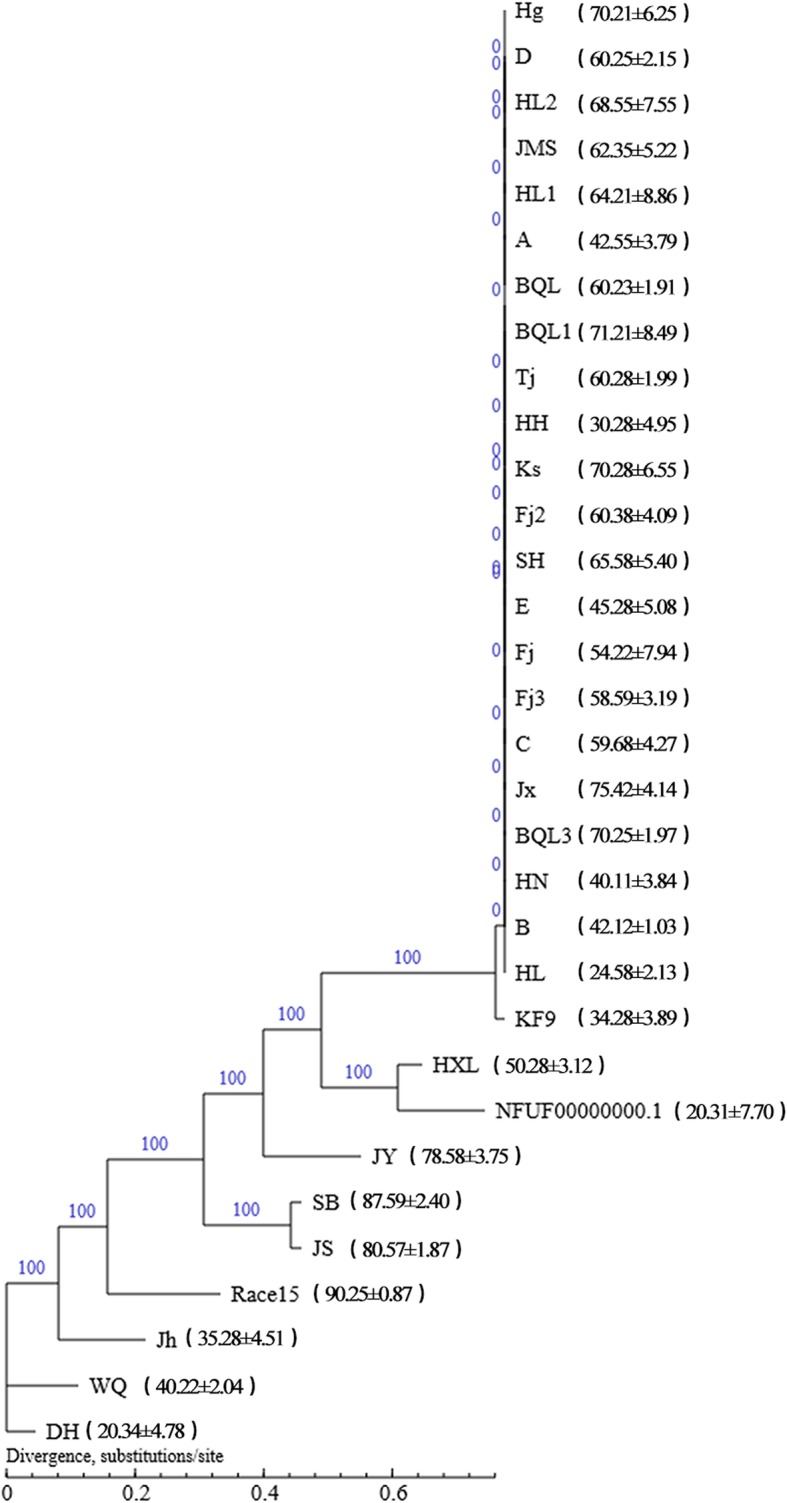
Phylogenic tree of the 30 *C. sojina* isolates constructed with 31,812 SNPs. Maximum Parsimony and the “hetequal” substitution model were used and 1000 bootstrap replicates were applied for tree building. The values in parentheses are the virulence values of each of the isolates. The number on each branch indicates the reliability of the branch. The branch length represents the size of the evolutionary distance, which was based on the average number of substitutions per nucleotide

Most isolates were collected from sites in the main soybean producing area in Northeast China, the region in China were FLS disease is the most serious. DH, JH and WQ were collected from Jilin province. The soybean planting areas here are relatively smaller than that in Heilongjiang province where the other isolates were collected. The phylogenetic tree revealed that WQ and DH had isolated branches, and the remaining 30 isolates all grouped into a single large branch. At the same time, the virulence value of WQ and DH was low, as these two are closely related to the area of soybean resistance cultivars where FLS disease prevalence is lower, and the annual effective accumulated temperature is significantly higher than that of the other 30 isolates collected from Heilongjiang. All of these isolates have close ancestral evolutionary relationships, consistent with the geographical location of the samples collected. Race15 had the highest virulence and was most closely related to strains SB, JS and JY. The virulence values of these 3 isolates were also relatively high (second, third, and fourth, respectively), while the proximal isolates of these 4 isolates were not highly virulent (Fig. 3).

### Linkage disequilibrium (LD) decay analysis and the association between SNP and virulence

**Fig. 4:**
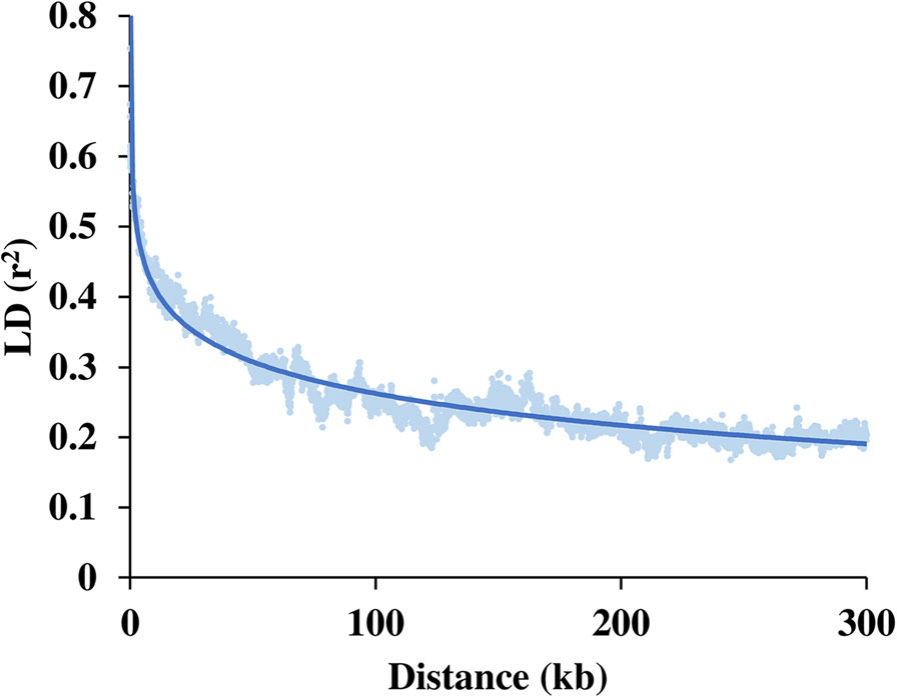
Genome-wide average linkage disequilibrium (LD) decay over physical distances based on 38,725 SNP markers. The half LD decay distance was 3.7 kb when the LD declined to 50% (r^2^ = 0.48) of its initial value. kb, kilo base pairs

Although the sexual stage of *C. sojina* has not been observed in either field or laboratory conditions, evidence suggests that the life cycle of *C. sojina* includes both a haploid stage and a sexual stage [17, 18]. SSR analysis has been used to analyze the mating-type ratio by others, and most of the populations showed a nearly 1:1 ratio [17]. In addition, we generated an LD decay plot and it showed that no significant decay of LD (r^2^ ≤ 0.1) was observed, but rather r^2^ decreased quickly to half of its maximum value at 3.7 kb physical distance (Fig. 4). This indicated that parts of the population were undergoing sexual recombination; thus, our GWAS data could be used for subsequent analysis.

Although our sample size was small, the filtered candidate genes could be used to help narrow the scope of virulence related genes. Plink analysis was used to identify a total of 1198 SNP sites associated with virulence values (*P* < 0.01). For all of the mutant sites that were filtered (31,812 SNP loci, with a minor allele frequency (MAF) less than 0.05 per loci before filtering, and 21,783 SNP loci after filtering, including homozygous mutation loci and heterozygous mutation loci), a total of 1198 SNP loci were identified to be associated with virulence (*P* < 0.01) (Additional file 12: Table S12). Among the significant loci with *P* < 0.01, 527 loci were located in genes, and 236 genes included at least one SNP loci. Of these 236 genes (Additional file 13: Table S13), there were 17 genes annotated in the PHI database, including at least one gene associated with a SNP, 7 genes annotated in the CAZy database, 9 genes predicted to be secretory proteins, and 26 genes predicted to be secondary metabolic processes (Additional file 14: Table S14). A total of 18 genes in the secondary metabolism library were predicted as Nonribosomal peptides (NRPs). NRPs are a class of peptide secondary metabolites usually produced by microorganisms like bacteria and fungi and are a very diverse family of natural products with an extremely broad range of biological activities. They are often toxins, siderophores, or pigments.

**Fig. 5:**
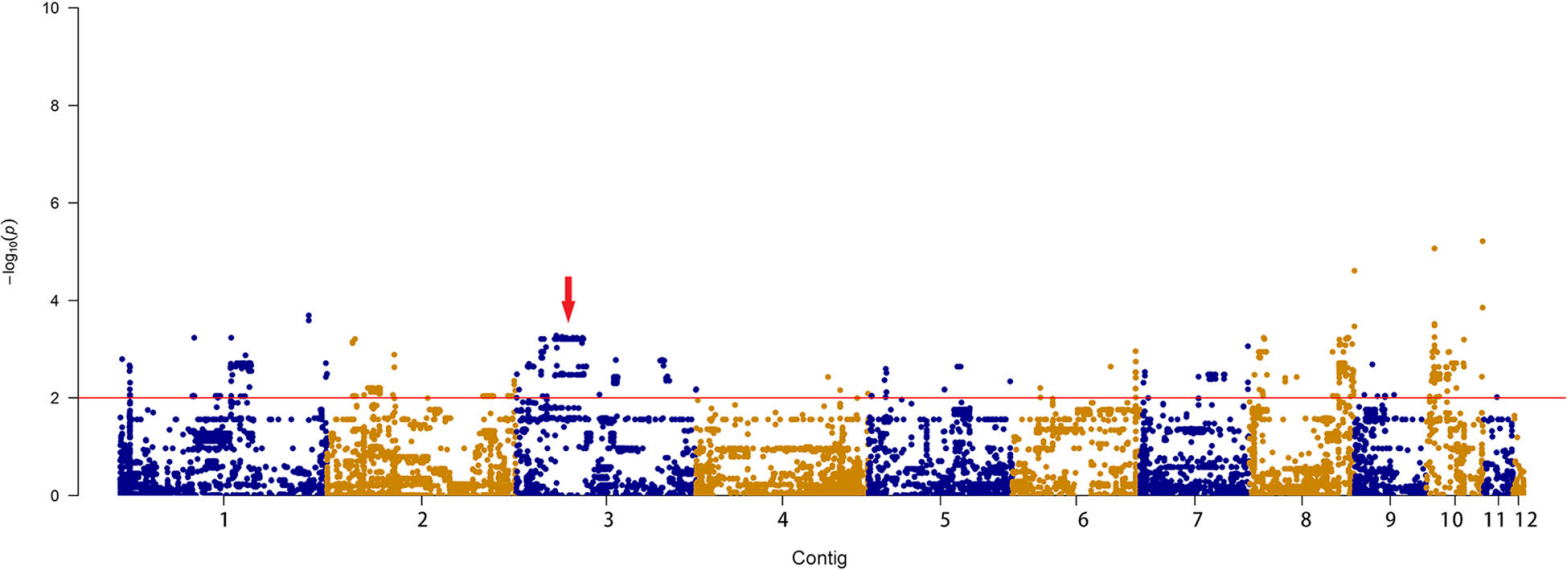
Manhattan plot of genome-wide association analysis (GWAS) of SNPs and virulence values. Dots above the red dashed line show the genome-wide significance at a stringent threshold of -log (0.01). The different colors indicate plots for different contigs, which follow the order: contig 1 – contig 12

Genomic association analysis of SNP and virulence value could be intuitively described using a Manhattan diagram. It can be seen from Fig. 5 that Contig3’s SNP sites are relatively concentrated and may be a candidate region for virulence gene association studies. There were 86 genes in the Contig3, including at least one SNP locus, of which FGSG09408 was worthy of attention, as it has been confirmed to play a role in the establishment of polarized growth and was up-regulated at 2 and 8 h, times when cell division and cytokinesis are activated during germination in *Fusarium graminearum* [30]. We used Qualimap to analyze the copy number of each sample, and our results showed that the coverage of contigs in each sample was consistent, meaning that there was no copy number variation.

### Genes correlated with virulence by differential counting of NSY SNPs

NSY SNPs have a more direct effect on the pathogenicity of fungi [8]. In the genomes of the 32 different isolates, a total of 3441 genes had at least one NSY mutation, and 485 genes had accumulated more than 50 NSY SNPs. There were 78 genes annotated in the PHI database, enzyme database (CAZymes), and predicted as Secretory Protein and secondary metabolic processes, respectively (Additional file 15: Table S15).

### Genes with different amounts of NSY SNPs between high and low virulence isolates

We examined genes with significant differences in the number of NSY SNPS between low-virulence and high-virulence samples, as these genes were most likely related to virulence. First, the virulence values of the 32 isolates were sorted from high to low. 11 isolates with virulence values more than 65 were classified as the high virulence group, and 11 isolates with virulence values less than 50 were classified as the low virulence group. A Wilcoxon rank sum test revealed that 69 genes had significant (*P* < 0.05) differences in the counts of NSY mutations between the low and high virulence groups. Of these mutations, 2 genes (5.8%) were annotated in the PHI database, 3 genes were annotated in CAZy, while 3 genes were predicted to be Secretory Protein candidate effectors. Collectively, 7 genes were speculated to be involved in secondary metabolic processes (Table 3).

**Table 3:** Number of NSY SNP mutations in 11 virulence related genes in high and low virulence groups

Gene ID	Locus	W^b^	*P* value	PHI	Secretory Protein	CAZy	Secondary Metabolism type
A08664	Contig5:2595828:2597615:−	87.5	0.038	*Fusarium graminearum* unaffected pathogenicity	NA^a^	NA	NA
A05514	Contig3:1656954:1661119:+	82.5	0.031	Fusarium graminearum reduced virulence	NA	NA	NA
A05389	Contig3:1317556:1320133:−	88	0.014	NA	YES	alpha;-L-rhamnosidase	NA
A05455	Contig3:1496013:1497815:+	82.5	0.031	NA	YES	NA	other
A08677	Contig5:2634601:2637864:+	87.5	0.038	NA	YES	alpha;-glucosidase	nrps
A00784	Contig1:3188237:3189785:−	82.5	0.031	NA	NA	Cellobiose dehydrogenase	NA
A05375	Contig3:1277694:1288561:−	82.5	0.031	NA	NA	NA	nrps
A05382	Contig3:1304494:1305336:−	82.5	0.031	NA	NA	NA	nrps
A05384	Contig3:1310009:1310896:+	82.5	0.032	NA	NA	NA	nrps
A12105	Contig9:836803:839562:+	81	0.045	NA	NA	NA	nrps
A05450	Contig3:1483773:1487649:+	82.5	0.032	NA	NA	NA	other

^a^*NA* not applicable

^b^*W* Wilcoxon rank sum value

A heatmap of the 11 genes with differential NSY SNP counts (*P* < 0.05) between the low and high virulence groups is shown in Fig. 5. For each gene, the various counts of NSY SNPs across the 32 isolates are indicated by blue, gray, and red colors corresponding to values ranging from low to high. 11 genes had fewer NSY mutation sites in the high-virulence group and more NSY SNP sites in the low-virulence group.

### Identification of important candidate genes

**Fig. 6:**
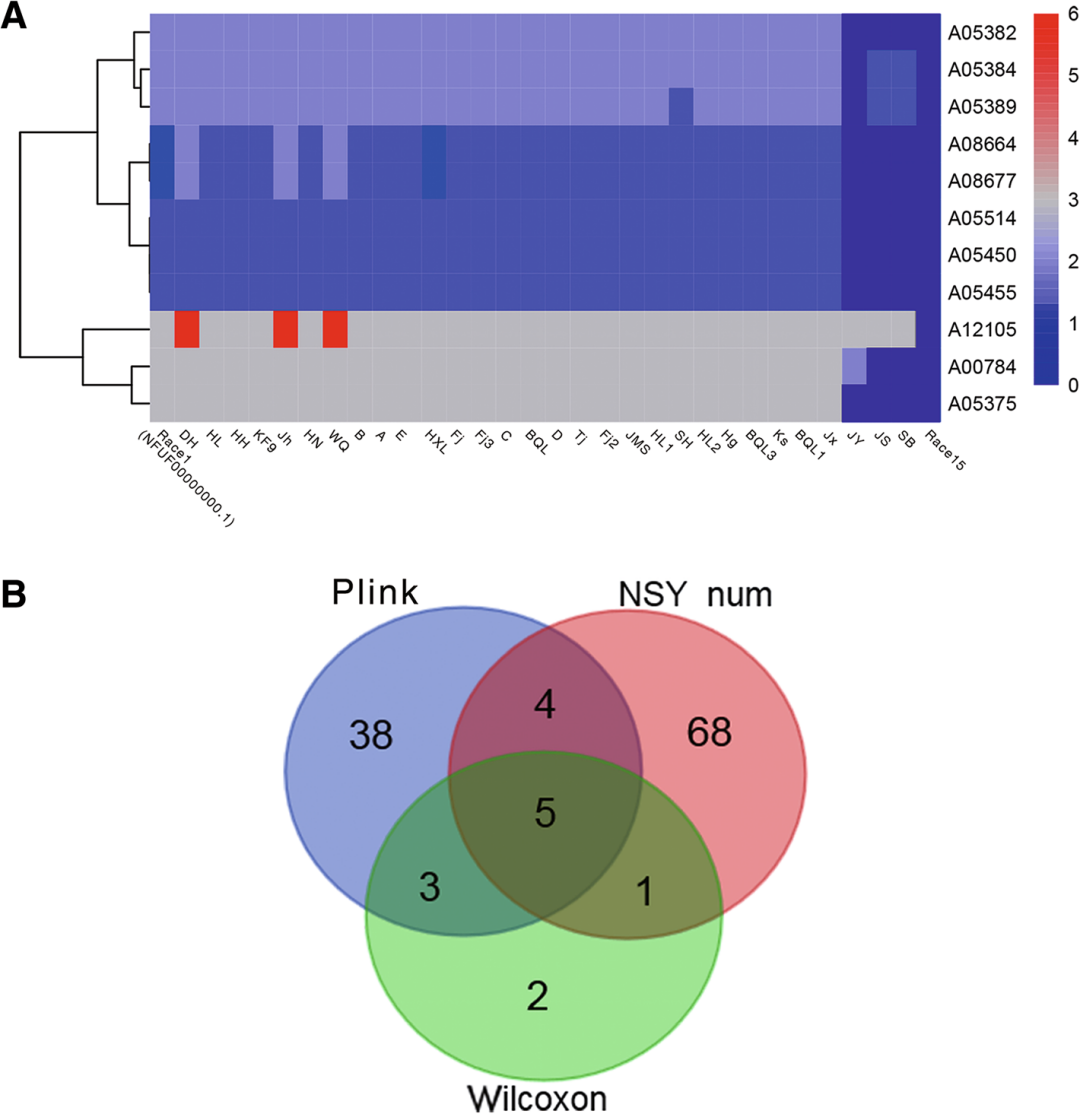
Heatmap of the 11 genes with differential non-synonymous (NSY) single nucleotide polymorphism (SNP) counts (*P* < 0.05), between the high and low-virulence groups, and a Venn diagram of the most critical virulence related genes identified using 3 methods. **a** Each row represents a gene and each column represents a sample. For each gene, the various counts of NSY SNPs across the 32 isolates are indicated by blue, gray, and red, corresponding to values ranging from low to high, respectively. **b** Green indicates the genes derived from Wilcoxon rank sum test (11 genes), Red indicates the counts of NSY SNP > 50 (78 genes), and Blue indicates the genes derived from SNP association

The most important candidate genes were identified by the intersection of these 3 kinds of genes, as shown in Fig. 6. Of the 5 identified key virulence related genes, 4 were in Contig 3 and 1 was in Contig 1, which was consistent with the virulence related areas detected in the Manhattan map (Table 4).

**Table 4:** Five virulence difference-related genes between Race15 and Race1

Gene ID	Locus	W^b^	*P* value	PHI	Secretory Protein	CAZy	Secondary Metabolism type
A05389	Contig3:1317556:1320133:−	88.00	0.0136802	NA^a^	YES	alpha;-L-rhamnosidase	NA^a^
A00784	Contig1:3188237:3189785:−	82.5	0.0313887	NA	NA	cellobiose dehydrogenase, etc	NA
A05375	Contig3:1277694:1288561:−	82.5	0.0307536	NA	NA	NA	nrps
A05382	Contig3:1304494:1305336:−	82.5	0.0307536	NA	NA	NA	nrps
A05384	Contig3:1310009:1310896:+	82.5	0.0316012	NA	NA	NA	nrps

^a^*NA* not applicable

^b^*W* Wilcoxon rank sum value

For instance, there were 3 SNP sites in the gene A05389, at contig3:1317647, contig3:1317841 and contig3:1319579. It was noted that in contig3:1317647, 4 high-virulent isolates (JS, JY, SB and Race15) shared the CC locus, whereas the other 28 isolates shared the AA locus that was absent in SH. In the contig3:1317841 and contig3:1319579 sites, the JS, JY and Race15 isolates shared the CC locus. SH and SB lacked this locus, while the other isolates shared the TT and AA loci in their respective contigs. After homology comparison, this locus was annotated as GH family 78 protein from *Bipolaris oryzae* (ATCC 44560), which was speculated as a secretory protein and annotated as α-L-Rhamnosidases in the CAZy database. α-L-Rhamnosidases catalyze the hydrolysis of terminal α-L-rhamnose residues from various carbohydrates. The catalytic domains in most of these enzymes belong to the GH78 and GH106 families of GHs [31].

There were 2 SNP sites in the gene A00784, at contig1:3188987 and contig1:3189373. In addition to JS, SB and Race15 shared the GG genotype, while the other isolates shared the CC locus that was absent in isolate SB. A00784 is located on Contig 1 and was speculated as an oxidoreductase in the CAZy database. By homology comparison, this was speculated as Glucose oxidase in *Cercospora beticola*. Glucose oxidases were speculated to specifically oxidize β-D-glucose to gluconic acid and hydrogen peroxide [32]. It has been clearly demonstrated that the hydrogen peroxide generated by glucose oxidases can inhibit the growth of pathogens [33]. To date, glucose oxidase genes have been cloned from *Penicillium expansum, Penicillium variabile* and *Talaromyces flavus* [34].

A05375 had 3 SNP sites, at contig3:1279311, contig3:1280051 and contig3:1280961. After homology comparison, this was annotated as non-ribosomal peptide synthetase 8 (NRPS8) from *Bipolaris maydis*. In contig3:1279311, JY, JS, and Race15 shared the GG locus, while the other isolates shared the TT homozygous genotype that was absent in isolate SH. In contig3:1280051, JS, SB, JY, and Race15 shared the TT locus, and the other isolates shared the GG locus. In contig3:1280961, JS, JY, SB, and Race15 shared the AA genotype and the other isolates shared the GG genotype that was absent in isolate SH.

A05382 is located in Contig 3 with 3 SNP sites, at contig3:1304880, contig3:1305120, and contig3:1305142, respectively. In contig3:1304880, in addition to the four isolates (JS, JY, SB and Race15) who shared the CC locus, the other isolates shared the AA locus. In contig3:1305120, JS, JY, SB and Race15 had the TT genotype, while the other isolates were CC. In contig3:1305142, JS, JY and Race15 were GG, and the other isolates were AA, which was absent in isolate SB. After homologous comparison, this locus was annotated in *Cercospora beticola* as O-acetyl-ADP-ribose deacetylase MACROD1 (CB0940–03553).

A05384 is located in Contig 3. JY, JS, SB and Race15 shared the TT genotype, while the others shared the CC genotype that was absent in isolate SH. After homology comparison, this locus was annotated as hypothetical protein (CB0940–03556) in *Cercospora beticola* with 1 SNP site.

It can be seen from the above results that the highly virulent isolates Race15, SB, JS, and JY had the same mutation sites in the above five genes, including SNPs or deletions. Considering their closeness in the phylogenetic tree and their putative shared ancestral state, some genes with the same mutation site may be related to species rather than virulence. Our study was designed simply to provide a useful reference for the screening of functional candidate genes, narrowing down the workload for subsequent analyses.

## Discussion

In this study, we sequenced the genome of Race15, a strain that has overcome soybean cultivars resistant to Race1. The assembled genome sequence was 40.12 Mb, with an N50 length of 4.9 Mb and a total of 601,794 PacBio reads being generated. Nowadays, the main strategy to unravel the *C. sojina* infection mechanisms has been to obtain its genome information. The genomes of Race1 [21] (China), FLS21 [3] (USA), and S9 [19] (USA) have been sequenced. By comparing the components of the genome, we found that the genome size, gene number, and gene total length of Race15 was close to that of Race1, and the genome size of the Chinese strains were significantly larger than those of the American FLS21 and S9 strains (Table 2). We speculate that this may be related to either sequencing precision or the environmental adaptability of these Chinese strains. In general, the larger a genome, the more likely it is to contain genetic information about metabolically related genes and stress-tolerance genes [35].

Comparative genomics revealed that although the Race15 and Race1 genomes have a large number of homologous genes, they have undergone a large number of genomic structural changes during evolution. All of these results were largely consistent with the EST-SSR genotype analysis, which showed that Race1 was close to Race15 on a phylogenetic tree of the tested isolates. Further, the pathogenic reaction patterns of Race1 and Race15 were similar too [13]. Coevolution of fungi with plant hosts has led to gene mutation in pathogens when resistance genes in host populations increases the selective pressure on the pathogen [36]. Since the environment of the Race15 and Race1 acquisition areas was basically the same, the differences have been presumed to be caused by the resistant cultivars, because the resistant cultivars grown in China have all been resistant to Race1 to date. Therefore, during the arms race between host and pathogen, the host’s response has caused a change in the dominant strain, in effect “selecting for” the Race15 strain.

Core genes and specific genes are likely to correspond to the commonness and characteristics of a sample, respectively, which can then be used as the basis for the study of functional differences between samples. In particular, the specific gene *Vtc4* from Race15 may be associated with high virulence, as this gene plays an important role for phosphate uptake in cryptococcal virulence. Deletion of the *Vtc4* gene, which encodes a polyphosphate polymerase, blocked the ability of *C. neoformans* to produce polyphosphate, and this influences fungal colonization of pulmonary tissue [28]. We know very little about the relationship between phosphate acquisition and virulence in fungal pathogens. In *U. maydis*, it is possible that *Vtc4* could participate in a protein complex with links to polarized growth and, in this case, negatively regulates filamentation [29].

At present, the separation frequency of Race15 is higher than that of Race1 in the soybean production areas of Northeast China. Synteny analysis also confirmed that the two races have high similarity, but there were also many differences. The isolates collected from more northerly region, such as JS, SB, JS and Race15, have high virulence, which has led to the progression of the original disease-free area into the present serious disease area. At the same time, the virulence values of WQ, JH and DH collected from the south of soybean producing areas in Northeast China were lower, and their genetic relationship are very close. These were all collected from Jilin province in the south of northeast Chinese soybean production area, this soybean area is much smaller than the northern Heilongjiang province. It indicates that the virulence of *C. sojina* population has obvious regional differences. The reason may be that isolates from different geographical regions are facing different geographical conditions and selection pressures, resulting in the directional selection of their genetic material. SB and JS are close to each other in the same branch and both of them are emerging soybean planting areas, while HXL and Race1 are in the same branch and both of them are perennial soybean planting areas. We speculate that this is related to the change of soybean planting areas from south to north in recent years. The changing of host cultivars and environments has forced the genetic and pathogenic variation of *C. sojina*, resulting in the production of new physiological strains [37]. This indicates that during the evolution of different isolates, a series of changes has taken place in the genome through the generation of SNPs, and the virulence of isolates over a certain period increases suddenly and lasts for a prolonged period of time. After mutual selection with the host, virulence gradually decreases and becomes stable.

Furthermore, the isolates were different based on differences in mapping rates, but the MP tree disguises these differences, making them appear as the same isolate. Thus, there appeared to be differences in the SNP numbers across isolates, but so many of them cluster with what seems like no distance whatsoever within the MP phylogenetic tree. Therefore, it was not very meaningful to use the whole genome of each isolate for differentiating strains. We applied resequencing technology as we did not obtain a whole genome sequence, due to the limitations of depth and our initial sequencing strategy. SNPs can accumulate and stabilize genetic variation, and the evolution of SNPs can accurately and directly reflect the evolution of a species over time. We used homozygous SNPs to construct a phylogenetic tree, as these represent stable genetic SNPs. The function of these is relatively important, because the number of homozygous SNPs was small, so the MP evolutionary tree difference was not obvious.

Five candidate genes were obtained by intersecting multiple virulence gene identification means. One of the genes, A05389 was speculated as α-L-Rhamnosidases in the CAZy database. At present, the research on α-L-Rhamnosidases in fungi is mainly focused on *Aspergillus*, *A. aculeatus* [38], *A. kawachii* [39], and *A. nidulans* [40]. The wood rot ascomycete *Xylaria polymorpha*, exhibits an α-L-Rhamnosidase that combines glycosyl hydrolase and esterase activities, helping this soft rot fungus to degrade lignocelluloses. All putative fungal members of this enzyme group, and the majority of the bacterial members, are lacking N-terminal signal peptides [41]. The A00784 gene was speculated as a glucose oxidase, and other studies have shown that fungal glucose oxidases can inhibit the activities of peroxidases, polyphenol oxidases and lipoxygenases in tomato fruits. Mutations of this enzyme in different varieties affects the activity of the enzyme, and thus, host resistance [42]. A05375 was speculated as NRPS8, which has been studied in *Aspergillus fumigatus* and is metabolized in its bioactive form. Studies have shown that it plays an important role in the biosynthesis of bioactive metabolites and may affect organism self-toxicity. Compared with other NRPSs, NRPS8 does not encode secretory peptides, but plays a more important structural role in germ tube formation [43]. A05382 was annotated as an O-acetyl-ADP-ribose deacetylase. This enzyme can remove the ADP-ribose portion of glutamic acid residues containing a single ADP ribose protein. O-acetyl-ADP-ribose is a signal molecule produced by the deacetylation of acetylated lysine residues, which are involved in the regulation of metabolic activity and epigenetic regulation [44].

Non-synonymous SNPs can be used for the detection of new fungal groups with potentially changed phenotypes, including drug resistance, increased virulence levels, and pathogenicity, all of which can influence disease severity [45]. This diversity is created by mutations affecting gene product structure and function [46–48]. Although our sample size was small, we compensated for this limitation by using three methods to identify virulence-related candidate genes (as annotated and predicted in four virulence-related databases). First, we identified the genes harboring at least one non-synonymous SNP associated with virulence using plink. Second, we identified genes with NSY SNPs > 50. Third, we identified genes with significant differences in the amounts of NSY SNPs between the high and low virulence groups via a Wilcoxon rank sum test. We then took the intersection of these three methods to further narrow the range of candidate genes. We found a large number of SNPs in each isolate, which meant that we had to refine our candidate gene selection in order further clarify the mechanism of virulence. Starting with our large number of SNPs, we found a correlation between SNPs and virulence differences between different isolates. Finally, the identified genes were the only candidates for virulence-related genes, which provided us an avenue for further study of virulence mechanisms and was helpful for reducing the workload for identifying resistant hosts. We also urge stronger focus on the CNVs that lead to phenotypic changes through changes in gene dose caused by both quantitative and qualitative effects, in that CNV association analysis may be easier for detecting pathogenic genetic variation. CNVs are known to provide phenotypic differences (sometimes due to elevated expression of specific genes) and it has been well documented that they can account for altered pathogenicity/virulence levels between fungal strains. Thus, they could offer some clues as to why Race15 possessed the highest virulence observed across the isolate set within this publication and why there was a synergistic effect when combined with the identified virulence factors using a GWAS approach. Our study was, however, the first to investigate the genomic variation of two *C. sojina* strains and was also the first to attempt genetic association analysis for the prediction of virulence genes in *C. sojina*.

## Conclusion

In summary, we sequenced Race15 of *C. sojina* and analyzed the comparative genome with respect to Race1. A large number of insertions and translocations were found, and some specific genes related to virulence were identified. Further, through GWAS, we identified five candidates by three different methods, and these candidate genes were speculated to be related to metabolic mechanisms and the biosynthesis of bioactive metabolites. Meanwhile, Race15 specific genes may be linked with high virulence. The genes highly prevalent in virulent isolates should also be proposed as candidates, even though they were not found in our SNP analysis. Future work should focus on using a larger sample size to confirm and refine candidate gene identifications and should study the functional roles of these candidates, in order to investigate their potential roles in *C. sojina* pathogenicity.

## Methods

### Isolate collection and virulence test

Between 1995 and 2016, soybean leaves with typical FLS lesions were collected from 32 fields in Northeast China’s provinces (Heilongjiang and Jilin) and their soybean area accounts for more than 77% of the total soybean area in China. In Heilongjiang province, 29 strains were collected from 29 individual fields, each field was separated by corn field and paddy field which were≈50 km, five to ten soybean leaves showing typical disease symptoms were collected from each field for *C. sojina* isolation. Additionally, 3 strains (DH, JH and WQ) were isolated from 3 individual fields in Jilin province. The specific coordinate information was shown in Table 1. FLS is not a quarantine disease, so no specific permits were required for sample collection in China. Sporulation was induced by incubating leaves in a plastic bag with moist towels at room temperature (approximately 24 °C). Spores were harvested with a flame-sterilized needle using a dissecting microscope and 10 spores transferred to V8 agar media containing ampicillin (100 μg/ml) [19].

The isolates of *C. sojina* used for whole genome sequencing and comparative genomics studies were isolated from the infected leaves of soybeans that were resistant to Race1 from Heilongjiang province of China. The isolates were identified as Race15 using Chinese differential cultivars. Thirty other isolates of *C. sojina* were collected from different ecological zones of soybean production in China for re-sequencing and were maintained as viable cultures in liquid nitrogen.

In order to identify the virulence of the 32 test isolates*,* the isolates were maintained on soybean stem agar and lima bean agar medium. Before spray inoculation, the conidia were scraped lightly from the agar in petri dishes using sterile water to make conidial suspensions. Subsequently, the conidial suspensions were filtered through sterilized multi-layer gauze and adjusted to a concentration of 6 × 10^4^ conidia mL^− 1^. Soybean seedlings were spray inoculated with the conidial suspensions at the V2–V3 growth stage of the soybean. One trifoliolate leaf per plant was inoculated with 0.3 mL of conidial suspension on the upper leaf surface. The inoculated seedlings were then placed in a humidity chamber at 26 to 28 °C for 72 h. Finally, 14 days after inoculation, the incidence level and disease index of each plant was calculated in order to determine the virulence of each isolate [12]. All virulence tests were repeated at least 3 times, with each isolate being used to inoculate at least 30 soybean plants. The plant materials used in the current study were collected from the Heilongjiang academy of agricultural sciences, which are public and available for non-commercial purpose.

### Fungal growth and genomic DNA extraction

The isolates of *C. sojina* that were used for genome sequencing and re-sequencing were cultured at 28 °C for 14 days on PDA medium, with a single spore being isolated 5 times. Then, the mycelium was removed from the culture medium and lapped in liquid nitrogen, and DNA was subsequently extracted using the CTAB method [49].

### Genome sequencing and assembly

The genome of *C. sojina* Race15 was sequenced using Single Molecule Real-Time (SMRT) technology and also 2 × 150 bp paired-end reads using an Illumina HiSeq 4000 by the Beijing Novogene Bioinformatics Technology Co., (Beijing, China). DNA libraries with 500 bp inserts and a mate-pair library with 5 kb inserts were used for de novo genome construction. SMRT Link v5.011 software (https://www.pacb.com/wp-content/uploads/SMRT_Link_User_Guide_v700.pdf) was used to filter low-quality reads, and then reads were assembled into scaffolds [50, 51]. Next, SOAP de novo [52] was used to map good quality paired reads onto the scaffolds and handle the reads for filling the gaps. For the re-sequencing of the 30 different isolates, each genome was sequenced with MPS (massively parallel sequencing) Illumina technology on the Illumina HiSeq 4000 platform using 2 × 150 bp paired-end reads. After sequencing, the Illumina PCR adapter reads and the low-quality reads from the paired-end and mate pair libraries were filtered as a quality control measure. Readfq.v10 (https://github.com/lh3/readfq) was used to filter low-quality sequences with the following parameters: -Q [40, 53], −N [10], −alen [15] --amis [3], and --dup, which filtered out reads containing Q < 53 and more than 40 bases, 10 N bases, adapters or duplications, respectively.

### Genome component prediction

The different components of the Race15 genome prediction, including coding genes, repetitive sequences and non-coding RNA, were then assigned. For fungi, the related coding genes were determined using the Augustus 2.7 program [53]. RepeatMasker v4.0.5 was used to predict interspersed repetitive sequences [54], and then tandem repeats were analyzed using TRF v4.07b (Tandem repeats finder) [55]. tRNA genes were predicted with tRNA scan-SE v1.31 [56]. rRNA genes were analyzed using rRNAmmer v1.2 [57]. sRNA, snRNA and miRNAs were predicted by BLAST against the Rfam database [58, 59].

### Genome function prediction and annotations

Gene functions were predicted by comparison with seven different databases. They were, respectively, GO [60–62] KEGG [61, 62], COG [63], NR [64], TCDB [65], P450 and Swiss-Prot [66]. The whole genome of Race15 was BLAST searched (E-value less than 1e-5, with a minimal alignment length percentage larger than 40%) against the above seven databases. The secretory proteins were predicted using the SignalP v4.1 [67] database to annotate whether there was a signal peptide, and TMHMM v2.0c [68] was used to annotate whether there was a transmembrane structure. Secondary metabolism gene clusters were predicted using antiSMASH v 2.0.2 (fungi) [69]. Pathogenicity gene and Carbohydrate-active enzymes were identified by searching against the Pathogen-Host Interactions database [70] and the Carbohydrate-Active enzymes database [24], respectively.

### Collinearity analysis and core-pan analysis

The MUMmer v3.23 software built-in tool nucmer, using the settings -minmatch/−l: [20] and --mincluster/−c: [65], was used to compare a target genome with a reference genome, and the large-scale collinearity between the genomes was then determined [71]. Next, LASTZ v 1.03.54 [72], set to --dup [50], −-inner [2000], −-gappedthresh [6000], and --identity [73], was used to compare regions, to confirm local location arrangement relationships, and to find regions with translocations, inversions and translocations + inversions [72, 74]. Their core genes and specific genes were analyzed using CD-HIT [73, 75], which allowed rapid clustering analysis of similar proteins with a threshold of 50% pairwise identity and 0.7 coverage.

### Genome alignment and SNP calling

Illumina reads from the 30 different isolates were aligned independently against the reference genome (Race15) assembly using BWA-MEM v0.7.8, with settings -t [4] and -k [32] and all other options set to default [76]. SAMTOOLS was used to count the coverage of the reference sequence and make explanations of the alignment results, which was also used for the detection of individual SNPs, insertions and deletions (< 50 bp) (Indel). The variation of each SNP was annotated in functional regions of the genome using SnpEff [77].

### Phylogenetic tree construction

Phylogenetic relationships of *C. sojina* collected from soybean production area in China that had different virulence were inferred using PAUP* based on SNP differences [78]. 31,812 SNPs were used to construct an evolutionary tree derived from 1000 bootstrap replicates. Finally, the phylogenetic tree was constructed using the maximum parsimony method variation of the Close-Neighbour-Interchange algorithm to analyze the evolutionary relationship between the isolates [79].

### LD decay and SNP statistical analyses

The pairwise measure of LD was estimated as squared allele frequency correlation (r^2^) values between pairs of intra-chromosomal markers with known chromosomal positions. LD decay was calculated using the PopLDdecay [80]. Plink software [81] was used to perform genome-wide association analysis (GWAS) using sample label swapping, with a max (T) permutation to compare the relationship between the virulence value and a SNP frequency [81, 82]. A Wilcoxon rank sum test was used to identify genes in the counts of non-synonymous (NSY) mutations between the high and low virulent groups. Finally, Heatmap.2 (https://cran.rproject.org/web/packages/gplots/gplots.pdf) was used to create heatmaps in order to display the number of NSY SNP mutations in virulence related genes [8].

## Supplementary information

**Additional file 1: Table S1.** Sequencing quality control results from Race15.

**Additional file 2: Table S2.** Coding gene prediction results from Race15.

**Additional file 3: Table S3.** Repeat sequence predictive statistical results.

**Additional file 4: Table S4.** Gene annotation/prediction statistics for Race15.

**Additional file 5: Table S5.** Genes from Race15 annotated in PHI.

**Additional file 6: Table S6.** Statistical of colinearity and coverage between Race15 and Race1.

**Additional file 7: Table S7.** Statistics of corepan genes between Race15 and Race1.

**Additional file 8: Table S8.** Genes of Race15 annotated in different databases.

**Additional file 9: Table S9.** General mapping information for the 30 *C. sojina* isolates.

**Additional file 10: Table S10.** Total number and type of SNPs in *C. sojina* isolates.

**Additional file 11: Table S11.** 31,812 SNP sites of the 32 isolates.

**Additional file 12: Table S12.** Results of genomic association analysis of SNP and virulence values.

**Additional file 13: Table S13.** Genes including at least one SNP.

**Additional file 14: Table S14.** Four virulence-related genes including at least one associated SNP.

**Additional file 15: Table S15.** Four virulence -related genes with the more than 50 non-synonymous SNPs.

## Data Availability

This Whole Genome Shotgun project has been deposited at NCBI under the accession PRJNA508859.

## Abbreviations

AA: Auxiliary activities enzyme
CBM: Carbohydrate-binding module
CE: Carbohydrate esterase
GH: Glycoside hydrolase
GT: Glycosyl transferase
GWAS: Genome-wide association analysis
MAF: Minor allele frequency
NRP: Nonribosomal peptides
NRPS: Non-ribosomal peptide synthase
NRPS8: Non-ribosomal peptide synthetase 8
NSY: Non-synonymous
PL: Polysaccharide lyase
QoI: Quinone outside inhibitor
SMRT: Single molecule real-time
SNP: Single nucleotide polymorphism
