# Supplementary material for: Comparative genomics and association analysis identifies virulence genes of Cercospora sojina in soybean
Source: BMC Genomics. 2020 Feb 19;21:172. doi: 10.1186/s12864-020-6581-5 (PMC7032006; doi:10.1186/s12864-020-6581-5)
Supplement: Supplementary file 1 — Additional file 1: Table S1. Sequencing quality control results from Race15. [file 12864_2020_6581_MOESM1_ESM.docx]

| Sample ID | Mean Concordance | Number of Reads | Number of Bases(bp) | Mean Read Length(bp) | N50 ReadLength(bp) |
| --- | --- | --- | --- | --- | --- |
| Race15 | 0.87 | 601794 | 6038283778 | 10033 | 13900 |

Table S1 Sequencing quality control results of Race15
