# Supplementary material for: Comparative genomics and association analysis identifies virulence genes of Cercospora sojina in soybean
Source: BMC Genomics. 2020 Feb 19;21:172. doi: 10.1186/s12864-020-6581-5 (PMC7032006; doi:10.1186/s12864-020-6581-5)
Supplement: Supplementary file 2 — Additional file 2: Table S2. Coding gene prediction results from Race15. [file 12864_2020_6581_MOESM2_ESM.docx]

Table S 2 Coding genes predicted results of Race15

| Sample ID | Type | Number(#) | Average length(bp) | Total length(bp) |
| --- | --- | --- | --- | --- |
| Race15 | tRNA | 200 | 129 | 25,882 |
| Race15 | 5s(denovo) | 1 | 116 | 116 |
| Race15 | 5.8s(denovo) | 0 | 0 | 0 |
| Race15 | 18s(denovo) | 1 | 1,799 | 1,799 |
| Race15 | 28s(denovo) | 0 | 0 | 0 |
| Race15 | sRNA | 2 | 216 | 432 |
| Race15 | snRNA | 13 | 135 | 1,765 |
| Race15 | miRNA | 0 | 0 | 0 |
