# Supplementary material for: Comparative genomics and association analysis identifies virulence genes of Cercospora sojina in soybean
Source: BMC Genomics. 2020 Feb 19;21:172. doi: 10.1186/s12864-020-6581-5 (PMC7032006; doi:10.1186/s12864-020-6581-5)
Supplement: Supplementary file 3 — Additional file 3: Table S3. Repeat sequence predictive statistical results. [file 12864_2020_6581_MOESM3_ESM.docx]

Table S3 Repeat sequence predictive statistical results

| Repbase stats | | | | |
| --- | --- | --- | --- | --- |
| Type | Number(#) | Total Length(bp) | In Genome(%) | Average length(bp) |
| LTR | 1892 | 722089 | 1.8 | 395 |
| DNA | 777 | 212833 | 0.5305 | 278 |
| LINE | 1750 | 1210870 | 3.0184 | 721 |
| SINE | 3 | 189 | 0.0005 | 63 |
| RC | 15 | 1200 | 0.003 | 84 |
| Unknown | 5 | 318 | 0.0008 | 84 |
| Total | 4442 | 2140679 | 5.3362 | 501 |
| Tandem Repeat stats | | | | |
| Type | Number(#) | Total Length(bp) | In Genome(%) | Average length(bp) |
| TRF | 4846 | 373589 | 0.9313 | 77 |
| Minisatellite DNA | 3236 | 163490 | 0.4075 | 51 |
| Microsatellite DNA | 972 | 39195 | 0.0977 | 40 |
