# Supplementary material for: Comparative genomics and association analysis identifies virulence genes of Cercospora sojina in soybean
Source: BMC Genomics. 2020 Feb 19;21:172. doi: 10.1186/s12864-020-6581-5 (PMC7032006; doi:10.1186/s12864-020-6581-5)
Supplement: Supplementary file 4 — Additional file 4: Table S4. Gene annotation/prediction statistics for Race15. [file 12864_2020_6581_MOESM4_ESM.docx]

Table S4 Gene annotation/prediction statistics of Race15

| database | genes with annotation |
| --- | --- |
| nr | 9242 |
| SwissProt | 2937 |
| KEGG | 8678 |
| KOG | 2023 |
| TCDB | 410 |
| GO | 8423 |
| P450 | 160 |
| PHI | 680 |
| Secretory_Protein | 766 |
| CAZy | 340 |
| Secondary_Metabolism | 777 |
