# Supplementary material for: Comparative genomics and association analysis identifies virulence genes of Cercospora sojina in soybean
Source: BMC Genomics. 2020 Feb 19;21:172. doi: 10.1186/s12864-020-6581-5 (PMC7032006; doi:10.1186/s12864-020-6581-5)
Supplement: Supplementary file 6 — Additional file 6: Table S6. Statistical of colinearity and coverage between Race15 and Race1. [file 12864_2020_6581_MOESM6_ESM.docx]

Table S6 Statistical of colinearity and coverage between Race15 and Race1

| Statistics project | The numerical |
| --- | --- |
| Target_alligned(bp) | 37550554 |
| Target_length(bp) | 40115976 |
| Target_coverage(%) | 93.6 |
| Query_alligned(bp) | 37548818 |
| Query_length(bp) | 40836407 |
| Query_coverage(%) | 91.95 |
| #blocks | 2915 |
