# Supplementary material for: Comparative genomics and association analysis identifies virulence genes of Cercospora sojina in soybean
Source: BMC Genomics. 2020 Feb 19;21:172. doi: 10.1186/s12864-020-6581-5 (PMC7032006; doi:10.1186/s12864-020-6581-5)
Supplement: Supplementary file 7 — Additional file 7: Table S7. Statistics of corepan genes between Race15 and Race1. [file 12864_2020_6581_MOESM7_ESM.docx]

Table S7 Statistics of corepan genes between Race15 and Race1

| Statistics project | The numerical |
| --- | --- |
| All Gene(#): | 25258 |
| Pan Gene(#): | 10843 |
| Core Gene(#): | 10,356 |
| Dispensable Gene(#): | 487 |
| Strain Specific Gene: |  |
| Race15： | 245 |
| Race1: | 274 |
