# Supplementary material for: Comparative genomics and association analysis identifies virulence genes of Cercospora sojina in soybean
Source: BMC Genomics. 2020 Feb 19;21:172. doi: 10.1186/s12864-020-6581-5 (PMC7032006; doi:10.1186/s12864-020-6581-5)
Supplement: Supplementary file 9 — Additional file 9: Table S9. General mapping information for the 30 C. sojina isolates. [file 12864_2020_6581_MOESM9_ESM.doc]

**Table S9** General mapping information for the 30 *C. sojina* isolates.

| **Query name** | **Total base** | **Total map base** | **Map rate** | **Avg depth** | **% of reference Covered by the reads** |
| --- | --- | --- | --- | --- | --- |
| Tj | 2,303,103,300 | 1,196,826,891 | 51.97 | 30 | 99.27 |
| B | 7,546,265,700 | 7,243,955,651 | 95.99 | 51 | 99.35 |
| C | 2,002,426,500 | 1,942,053,397 | 96.99 | 48 | 99.34 |
| HL2 | 1,905,022,200 | 1,841,580,158 | 96.67 | 46 | 99.33 |
| Fj | 5,734,463,100 | 5,538,811,294 | 96.59 | 41 | 99.34 |
| A | 2,054,731,200 | 1,982,427,859 | 96.48 | 49 | 99.34 |
| HH | 2,113,093,200 | 2,020,703,541 | 95.63 | 50 | 99.34 |
| DH | 2,267,938,500 | 2,154,727,835 | 95.01 | 54 | 99.63 |
| HN | 2,297,485,500 | 2,203,586,223 | 95.91 | 55 | 99.35 |
| Fj2 | 2,218,337,100 | 2,143,561,520 | 96.63 | 53 | 99.35 |
| E | 1,907,600,400 | 1,631,883,103 | 85.55 | 41 | 99.33 |
| Hg | 1,782,962,100 | 1,077,561,790 | 60.44 | 27 | 99.25 |
| HXL | 1,775,423,700 | 1,694,687,263 | 95.45 | 42 | 99.31 |
| KF9 | 1,966,969,800 | 1,9143,60,785 | 97.33 | 48 | 99.34 |
| WQ | 1,821,820,800 | 1,709,726,029 | 93.85 | 43 | 99.27 |
| SB | 1,862,394,300 | 446,666,637 | 23.98 | 11 | 99.2 |
| HL | 5,852,183,400 | 4,500,754,312 | 76.91 | 38 | 99.29 |
| Jh | 1,800,119,700 | 1,712,559,074 | 95.14 | 43 | 99.68 |
| BQL | 5,413,762,800 | 5,202,584,735 | 96.1 | 40 | 99.31 |
| JS | 1,525,366,500 | 1,435,717,291 | 94.12 | 36 | 99.5 |
| Jx | 1,680,214,500 | 1,615,796,559 | 96.17 | 40 | 99.33 |
| JY | 1,761,583,500 | 1,684,287,267 | 95.61 | 42 | 99.33 |
| Ks | 1,832,224,800 | 1,761,024,785 | 96.11 | 44 | 99.34 |
| BQL1 | 1,801,489,500 | 1,735,215,077 | 96.32 | 43 | 99.33 |
| BQL3 | 1,917,390,600 | 1,856,934,956 | 96.85 | 46 | 99.34 |
| SH | 1,830,652,800 | 480,546,186 | 26.25 | 12 | 99.05 |
| HL1 | 1,813,525,800 | 1,137,812,612 | 62.74 | 28 | 99.27 |
| D | 3,978,773,100 | 2,948,202,579 | 74.1 | 20 | 99.2 |
| Fj3 | 1,798,921,800 | 1,731,246,903 | 96.24 | 43 | 99.33 |
| JMS | 1,695,906,600 | 1,008,172,920 | 59.45 | 25 | 99.25 |
