# Supplementary material for: Comparative genomics and association analysis identifies virulence genes of Cercospora sojina in soybean
Source: BMC Genomics. 2020 Feb 19;21:172. doi: 10.1186/s12864-020-6581-5 (PMC7032006; doi:10.1186/s12864-020-6581-5)
Supplement: Supplementary file 10 — Additional file 10: Table S10. Total number and type of SNPs in C. sojina isolates. [file 12864_2020_6581_MOESM10_ESM.docx]

**Table S10** Total number and type of SNPs in *C. sojina* isolates.

| **Sample** | **#Non syn** | **#Syn** | **#Intergenic** | **Ts** | **tv** | **ts/tv** | **Het** | **Hom** | **Total** | **Density (SNP/Kb)** |
| --- | --- | --- | --- | --- | --- | --- | --- | --- | --- | --- |
| A | 2973 | 2393 | 8121 | 9145 | 4342 | 2.11 | 1009 | 12478 | 13487 | 0.34 |
| B | 2973 | 2399 | 8132 | 9122 | 4382 | 2.08 | 1023 | 12481 | 13504 | 0.34 |
| BQL | 2956 | 2387 | 8064 | 9101 | 4306 | 2.11 | 1024 | 12383 | 13407 | 0.33 |
| BQL1 | 2967 | 2390 | 8108 | 9125 | 4340 | 2.1 | 1027 | 12438 | 13465 | 0.34 |
| BQL3 | 2972 | 2393 | 8148 | 9156 | 4357 | 2.1 | 1029 | 12484 | 13513 | 0.34 |
| C | 2956 | 2378 | 8116 | 9117 | 4333 | 2.1 | 1008 | 12442 | 13450 | 0.34 |
| D | 2916 | 2297 | 7889 | 8884 | 4218 | 2.11 | 991 | 12111 | 13102 | 0.33 |
| DH | 1901 | 1466 | 5582 | 6063 | 2886 | 2.1 | 1172 | 7777 | 8949 | 0.22 |
| E | 2970 | 2371 | 8150 | 9129 | 4362 | 2.09 | 1077 | 12414 | 13491 | 0.34 |
| Fj | 2969 | 2384 | 8137 | 9150 | 4340 | 2.11 | 1062 | 12428 | 13490 | 0.34 |
| Fj2 | 2964 | 2396 | 8139 | 9155 | 4344 | 2.11 | 990 | 12509 | 13499 | 0.34 |
| Fj3 | 2955 | 2376 | 8077 | 9100 | 4308 | 2.11 | 995 | 12413 | 13408 | 0.33 |
| HH | 2975 | 2398 | 8107 | 9143 | 4337 | 2.11 | 1020 | 12460 | 13480 | 0.34 |
| HL | 2938 | 2376 | 8060 | 9073 | 4301 | 2.11 | 1011 | 12363 | 13374 | 0.33 |
| HL1 | 2955 | 2342 | 8027 | 9027 | 4297 | 2.1 | 1010 | 12314 | 13324 | 0.33 |
| HL2 | 2985 | 2395 | 8128 | 9161 | 4347 | 2.11 | 1050 | 12458 | 13508 | 0.34 |
| HN | 2975 | 2400 | 8128 | 9154 | 4349 | 2.1 | 1014 | 12489 | 13503 | 0.34 |
| HXL | 2623 | 2126 | 7706 | 8442 | 4013 | 2.1 | 1566 | 10889 | 12455 | 0.31 |
| Hg | 2936 | 2357 | 7956 | 8996 | 4253 | 2.12 | 1010 | 12239 | 13249 | 0.33 |
| JMS | 2928 | 2353 | 7929 | 8952 | 4258 | 2.1 | 960 | 12250 | 13210 | 0.33 |
| JS | 2139 | 1726 | 6625 | 7077 | 3413 | 2.07 | 1386 | 9104 | 10490 | 0.26 |
| JY | 2471 | 1949 | 7554 | 8109 | 3865 | 2.1 | 1078 | 10896 | 11974 | 0.3 |
| Jh | 2044 | 1519 | 6087 | 6488 | 3162 | 2.05 | 1337 | 8313 | 9650 | 0.24 |
| Jx | 2965 | 2400 | 8065 | 9106 | 4324 | 2.11 | 1027 | 12403 | 13430 | 0.33 |
| KF9 | 2968 | 2374 | 8153 | 9160 | 4335 | 2.11 | 1051 | 12444 | 13495 | 0.34 |
| Ks | 2998 | 2401 | 8181 | 9204 | 4376 | 2.1 | 1079 | 12501 | 13580 | 0.34 |
| SB | 2196 | 1633 | 6227 | 6781 | 3275 | 2.07 | 1531 | 8525 | 10056 | 0.25 |
| SH | 2897 | 2242 | 7576 | 8725 | 3990 | 2.19 | 1017 | 11698 | 12715 | 0.32 |
| Tj | 2931 | 2379 | 7986 | 9019 | 4277 | 2.11 | 1009 | 12287 | 13296 | 0.33 |
| WQ | 2173 | 1747 | 6717 | 7227 | 3410 | 2.12 | 1418 | 9219 | 10637 | 0.27 |
| Race 1 | 2306 | 1806 | 6580 | 7097 | 3595 | 1.97 | 0 | 10692 | 10692 | 0.27 |
